# Optimizing the impact of a novel spatial repellent on malaria incidence in Western Kenya

**DOI:** 10.1017/S095026882610171X

**Published:** 2026-06-08

**Authors:** Tiffany Huwe, Cristian Koepfli, Eric O. Ochomo, John E. Gimnig, Nicole L. Achee, John P. Grieco, Bernard Abong’o, Vincent Moshi, Alex Perkins, Sean M. Moore

**Affiliations:** 1 University of Notre Dame, USA; 2 Biological Sciences, University of Notre Dame, USA; 3 Centre for Global Health Research, Kenya Medical Research Institute, Kenya; 4 Vector Group, Liverpool School of Tropical Medicine, Liverpool, UK; 5 Centers for Disease Control and Prevention, USA

**Keywords:** malaria, Anopheles, spatial repellent, spatial emanator, vector control, insecticide

## Abstract

Progress in reducing global malaria incidence has slowed in recent years, demonstrating the need for new vector control tools to complement existing interventions in reaching global malaria control targets. Spatial repellents (SRs) can reduce pathogen transmission by altering the behaviors of Anopheles spp. mosquito vectors. A recent cluster-randomized control trial in western Kenya found that SRs reduced first-time infections by 33.4% in a highly-endemic setting with substantial insecticide resistance and high coverage of insecticide treated nets. We modeled the likely impact of the SR intervention in this setting under different deployment strategies, with the goal of identifying the best overall and per-product strategies. Continuous monthly SR deployment with 100% coverage caused the greatest (45.1%) reduction in infections versus no SR, although some seasonal approaches averted more infections per SR product. Six months of SR use starting in October caused a 36.4% reduction in infections and eight months starting in September reduced infections by 44.7%. This study identified optimum and efficient SR use strategies and highlights the importance of SR protection during periods of low and increasing transmission. When resources are limited, the coverage, deployment strategy, and timing of SR deployment all play a key role in maximizing product impact.

## Introduction

The global incidence of malaria declined steadily from 2000 to 2015 largely due to the increased use of long-lasting insecticide-treated bed nets (LLINs) and indoor residual spraying (IRS) [1]. However, progress slowed in the last decade, even before programme interruptions related to COVID-19 allowed global malaria case numbers to increase for the first time this millennium [2]. The WHO estimates that there were 263 million cases of malaria globally in 2023, highlighting the need to develop novel vector control tools to supplement existing interventions and promote continued progress towards disease control and elimination [3]. Spatial repellents (SRs; also known as spatial emanators) are one such promising new product class that were recently conditionally recommended by the WHO Vector Control Advisory Group for use in the control and prevention of malaria [4].

SRs release volatile pyrethroid chemicals into the air, which interfere with mosquito vectors’ host-seeking and feeding behaviours, thereby reducing human-vector contact [5–7]. In addition to these behavioural impacts, SRs can also increase mosquito mortality depending on the dose exposure [8–12]. Such products could offer protection in and around households where LLINs are not used, as well as in the evening and early morning hours when biting may occur but people are not protected by LLINs [13–16]. Entomological studies have shown that transfluthrin spatial repellents are effective in reducing host-seeking, landing, and blood-feeding of *Aedes* and *Anopheles* vectors, including those that are resistant to other pyrethroids (i.e., permethrin and deltamethrin) commonly used on LLINs [11, 12, 17–19]. A small but growing body of research indicates that SRs are protective against transmission of arboviruses and malaria [18, 20–22]. A recent cluster randomized field trial in western Kenya reported 33.4% protective efficacy of transfluthrin SRs against first-time malaria infection after continuous 84% product coverage for 1 year [22]. These results were achieved in a theoretically difficult setting, where *P. falciparum* prevalence is high (50% positive by qPCR), vectors have a high rate of permethrin-resistance, and residents already have partial protection from near-universal use of pyrethroid plus piperonyl butoxide (PBO) LLINs [22, 23]. A recent study found that *An. funestus* in Busia County, Kenya, had reduced 24-h mortality (70% and 87%) when exposed to 0.75% permethrin or 0.05% deltamethrin, respectively [24]. However, *An. funestus* mortality was 100% when permethrin or deltamethrin was combined with 4% PBO. An earlier study found even higher rates of resistance in Busia from 2009 to 2010, with 0.75% permethrin causing only 16% mortality in *An. gambiae s.s.* and 54% *An. gambiae s.l.* [25].

It is not well understood how various SR deployment strategies may affect the protective efficacy of the intervention, and field trials to evaluate various strategies would be logistically difficult, expensive, and time-consuming. Dynamic modelling offers an opportunity to evaluate disease control strategies in a less resource-intensive manner. Dynamic transmission models have been used to compare the likelihood of achieving malaria elimination in a particular setting under different intervention strategies [26], assess the ability of various control strategies to prevent reestablishment of malaria where the disease was recently eliminated [27], and evaluate the level of disease protection offered by chemoprevention with or without vaccination [28]. Furthermore, modelling studies of IRS and antimalarial drug administration suggest that campaign timing relative to seasonal transmission intensity can impact their effectiveness [29–31]. For example, one modelling study of a setting with seasonal rainfall and malaria transmission found that the maximum prevalence reduction was achieved by deploying IRS and drug administration during the peak transmission season [31].

Field trials indicate that SRs are effective in protecting against malaria with continuous use, but it is not understood how protection may vary for seasonal SR campaigns or at lower levels of product usage at the community level. In this study, a model of malaria transmission was calibrated to the conditions in western Kenya and used to evaluate the impact of various deployment strategies on the effectiveness of SRs against malaria incidence. The goal was to gain insights into the level of SR product coverage required to grant community protection and assess whether seasonal use may be appropriate where malaria transmission varies seasonally.

## Methods

### Study area and model details

**Figure 1.**
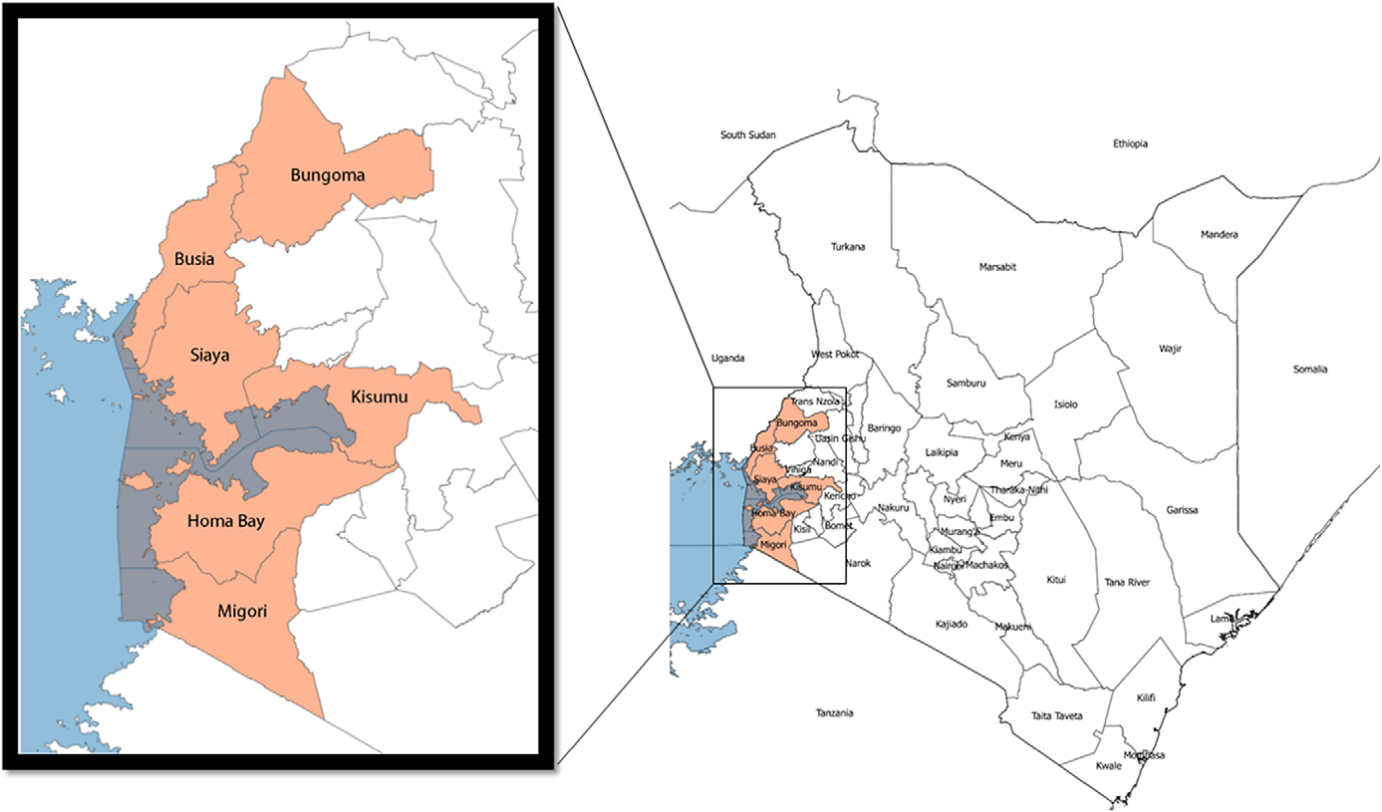
Map of Western Kenya showing the six counties included in our simulation studies. Field trials occurred in Busia county [22].

A model was set up to simulate malaria transmission dynamics in the lake-endemic region of western Kenya near Lake Victoria, a high transmission area where 27% of children aged 0–14 years tested microscopy positive for *Plasmodium* in 2015 [32] (Figure 1). Simulations were run using Epidemiological MODeling software (EMOD v2.21), a stochastic, agent-based model that simulates malaria transmission in a complex landscape of individual humans, vectors, *Plasmodium falciparum* parasites, and various malaria interventions [33]. The age distribution of human agents in the model was set to match observed demographics, and model humans experienced a surface area-dependent increase in biting risk with age. In each one-day time step, vectors progress through a decision tree where they may survive or succumb to natural mortality, attempt to feed on an animal or a human, attempt to feed indoors or outdoors, etc. according to a set of conditional probabilities. If an infectious vector succeeds in finding a host and feeding, it has a 50% chance of transmitting sporozoites to the host (before accounting for host immunity modifiers). If a vector takes a successful blood meal from an infectious host, it has a 30% chance of taking up mature gametocytes and becoming infected.

### Model calibration

We incorporated local climate and demographics data for six counties (Bungoma, Busia, Homa Bay, Kisumu, Migori, and Siaya) into the model (Figure 1). We calibrated the model using mosquito abundance data [34–37] and previously published parasite prevalence data from the 2015 Malaria Indicator Survey [38] to reproduce baseline epidemiological conditions in the region prior to the start of the field trials in Busia County. The initial model calibration step was conducted using a maximum likelihood approach via a gradient ascent iterative optimization algorithm [39]. After baseline data collection in the field trial, and prior to SR distribution, a mass distribution of new pyrethroid plus piperonyl butoxide (PBO) bed nets was conducted in the study area. Perhaps because of this PBO net distribution, incidence dropped from 3.2 infections/pp./yr. at baseline to 1.4 infections/pp./yr. in the placebo arm of the field study during the implementation phase [22]. Therefore, we estimated the impact of non-PBO bed nets using the baseline incidence rate and estimated the impact of PBO bed nets using the incidence rate in the placebo arm of the field trial.

EMOD includes parameters that allow spatial repellents to reduce the probability that a mosquito enters a house while host seeking [blocking effect] and the probability that a mosquito that does enter a house dies before attempting to feed (and before encountering a bed net if one is present in the house) [killing effect]. We estimated these two parameters by fitting the model with SR deployment to the observed 32.7% [CI 90%: 16.2%–46.0%] protective efficacy against first-time malaria infection [22]. For our main analysis, we sampled 50 blocking/killing parameter combinations with replacement based on their normalized likelihoods (Supplementary Figure S7). The mean blocking effect was 0.519 (IQR: 0.375–0.725), representing a 51.9% reduction in the probability of a host-seeking mosquito entering an SR-treated house. The mean killing effect was 0.473 (IQR: 0.313–0.607). This compares to a blocking effect of 0.8 and estimated killing effect of 0.45 for PBO bed nets. Additional details on each step of the calibration process are provided in the Supplementary Materials (SI Methods).

### Simulating SR use for malaria control

**Figure 2.**
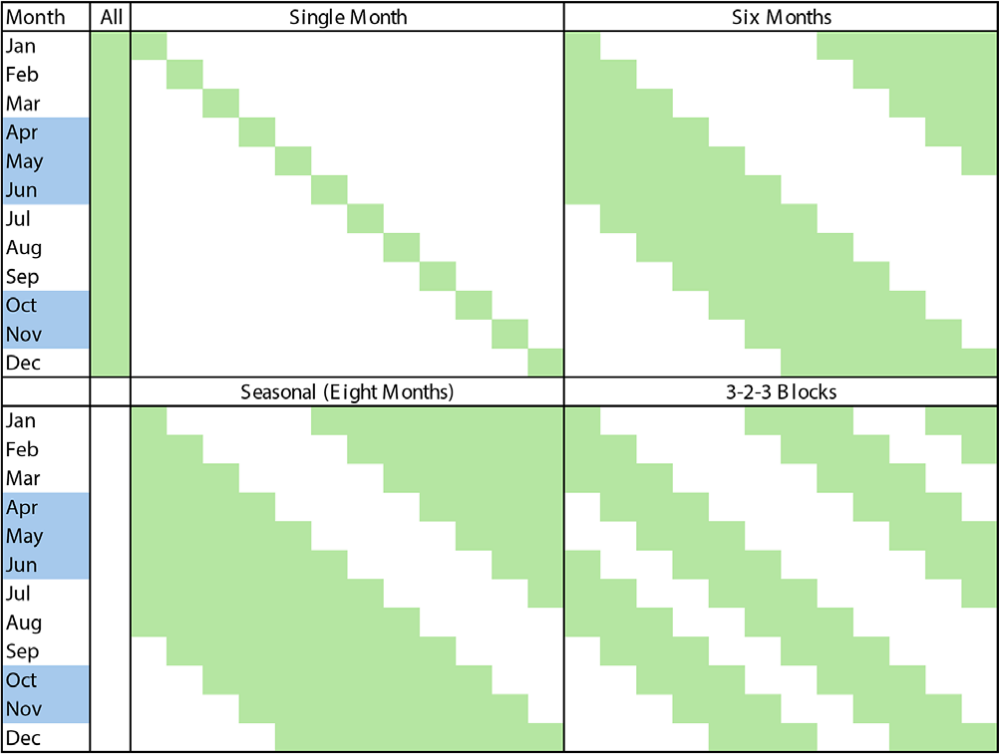
Seasonal SR deployment strategies. Each column represents one of the deployment schedules. Green blocks indicate that SRs are used during that month. Months in blue indicate the long and short rainy seasons. Figure 2. long description.

To investigate optimum use of spatial repellents, simulations were carried out with variable SR coverage and deployment schedules. A negative control scenario was simulated in which the only malaria interventions were treatment with an artemisinin-based combination therapy (ACT) when symptomatic and 95% coverage with PBO bed nets. Experimental scenarios included ACT and PBO bed nets and added spatial repellent products with 30 days of effectiveness. SR coverage ranged from 10% to 100%. SR deployment schedules included all months, a single month, six-month blocks, seasonal 8-month blocks, and a 3-2-3 schedule (two three-month blocks with a two-month gap in between, followed by a four-month gap) (Figure 2). We refer to these deployment schedules as Single-X, Half-X, Season-X, and Block-X, respectively, where X is the month when the schedule begins. The last scheme was designed to offer SRs during both rainy seasons with a break during the short dry period between them.

Each simulation included a four-year burn-in period with PBO bed net and ACT interventions, followed by 2 years during which SRs were added to the control toolkit. For each combination of SR coverage and distribution schedules, 50 stochastic realizations of the simulation were run using each of the 50 sampled SR parameter combinations. Model outputs are presented as medians and interquartile ranges across the 50 realizations for each scenario. The proportion change from the negative control scenario was calculated relative to the mean value from the 50 negative control realizations. The proportion of infections detectable by rapid diagnostic tests (RDTs) was calculated assuming a limit of detection of 100 parasites/uL blood [40, 41].

## Results

**Figure 3.**
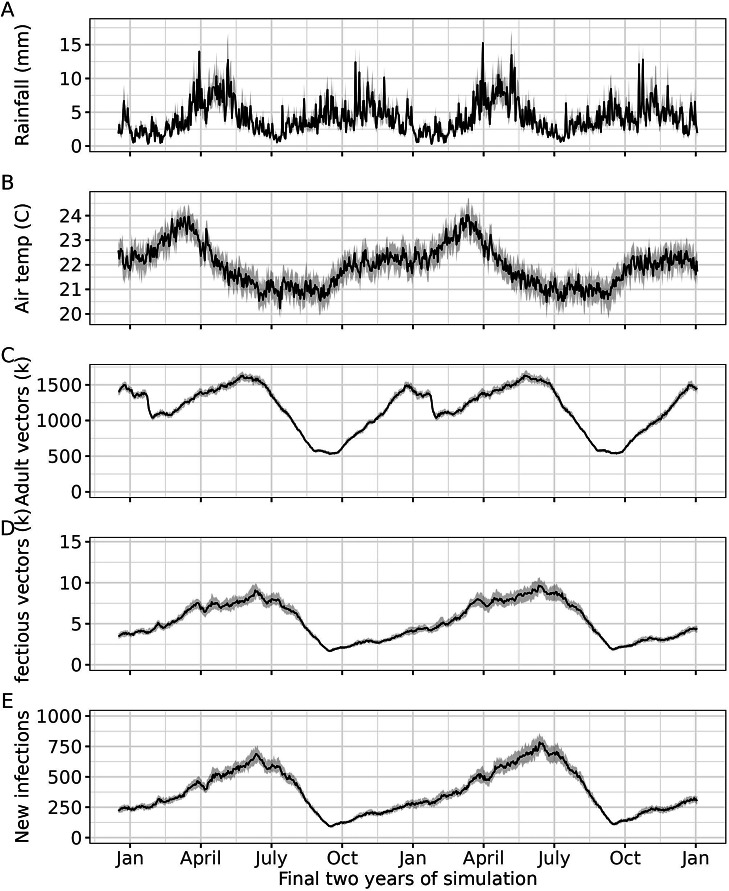
Model output data from the last 2 years of simulations with no spatial repellent (SR). Lines represent the median, and clouds represent the IQR. Figure 3. long description.

After the burn-in period, the negative control model with no SR displayed stable seasonal patterns (Figure 3). Two rainy periods occurred each year in April–June and October–November, and air temperature peaked in March prior to the onset of the first rainy season. The number of adult vectors in the population peaked in June and January following each rainy season; however, the numbers of infectious vectors and new infections exhibited just one peak per year in June. The steady increase in infectious vectors and new infections from October through June may be related to the parasite dynamics and human behaviour during this period of moderate and increasing temperatures.

**Figure 4.**
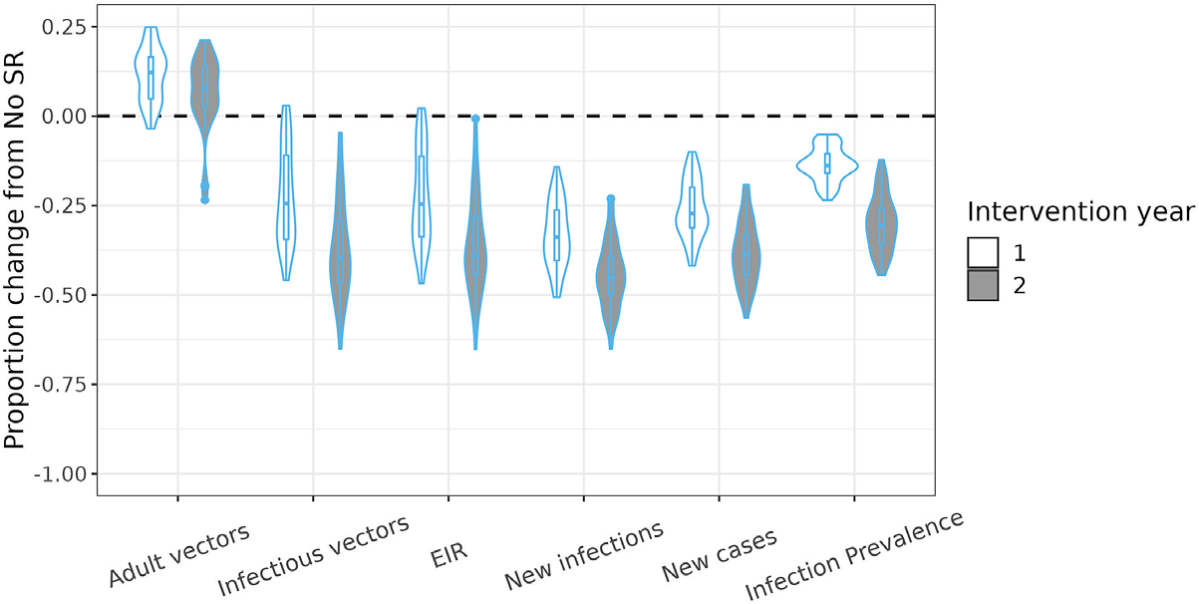
Proportional changes in output measurements from the No SR control in the first and second years of SR intervention when modelling All Months SR use with 100% coverage. Figure 4. long description.

SR use impacted numerous entomological and epidemiological measures (Figure 4). After 1 year of continuous SR use at 100% coverage, the model population had 13% more adult vectors (IQR: +5% to 17%), 24% fewer infectious vectors (IQR: −11% to 34%), and 24% lower EIR (IQR: −11% to 34%) than the model with no SRs. The increase in mosquito abundance accompanied by high SR coverage is likely the result of reduced mosquito contact with LLINs, which have a higher combined blocking and mortality effect than the SR product. The SR model also had 34% fewer new *Plasmodium* infections (IQR: −26%–40%), 27% fewer new malaria cases (IQR: −20% to 31%), and 13% lower infection prevalence (IQR: −11% to 16%) than the model with no SRs. In the second year, the effects of SR use became more pronounced. After 2 years of continuous SR use, the model population had 8% more adult vectors (IQR: +3%–14%), 40% fewer infectious vectors (IQR: −29% to 46%), and 39% lower EIR (IQR: −28% to 44%) than the model with no SRs. The second year SR model also had 45% fewer new *Plasmodium* infections (IQR: −39% to 47%), 39% fewer new malaria cases (IQR: −33% to 44%), and a 31% lower infection prevalence (IQR: −26% to 36%) than the model with no SRs.

**Figure 5.**
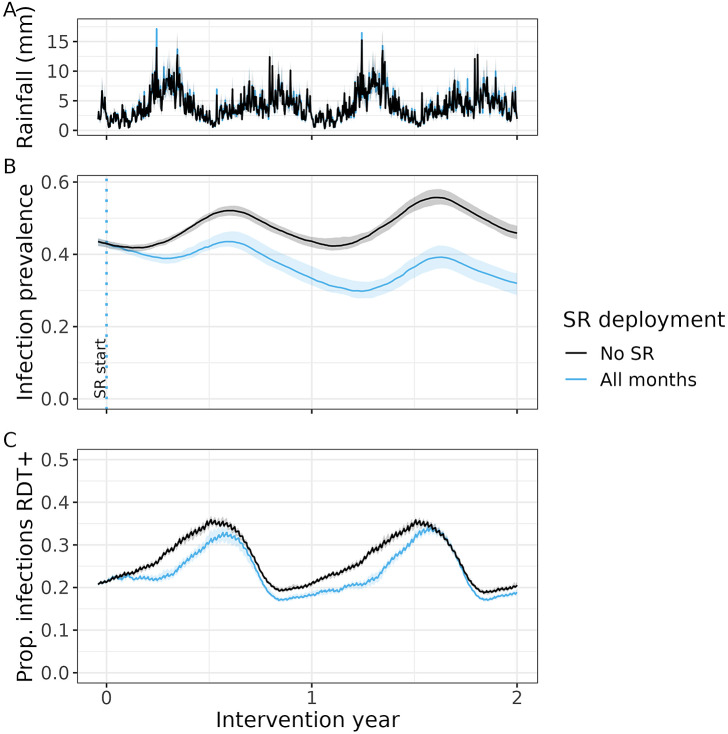
(a) Median rainfall (mm), (b) infection prevalence, and (c) proportion of infections detectable by RDTs over 2 years. The black line represents No SR, and the blue line represents the All Months SR deployment schedule. Figure 5. long description.

In the model with continuous SR deployment, *Plasmodium* infection prevalence maintained a seasonal pattern with peak prevalence in July of each year. Peak infection prevalence with SR use decreased from 44% in the first year to 39% in the second year, despite an increase in peak infection prevalence from 52% to 56% in the negative control (Figure 5). The largest absolute difference in infection prevalence between the SR and negative control models (17%) occurred in July of the second year of intervention. The proportion of infections detectable by RDTs followed a similar seasonal pattern, although peak detectability in the SR model was slightly lower (33%) and occurred about a month later than in the negative control (36%). Reduced RDT detectability in the SR model suggests that a smaller portion of *Plasmodium* infections reach higher densities where they are more likely to be infective to mosquitoes and become symptomatic malaria cases, especially in the first half of the year.

**Figure 6.**
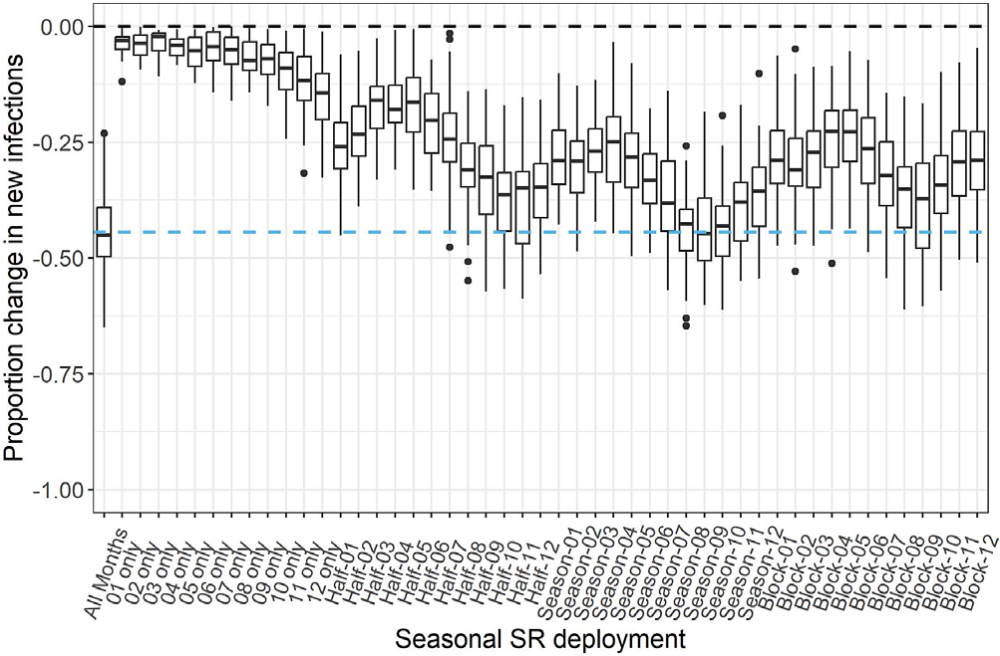
Proportional changes in new infections from the No SR control (black dotted line) in the second year of SR intervention when modelling various seasonal SR deployment schedules, each with 100% coverage. The blue dotted line indicates the average proportional change for All Months. Figure 6. long description.

A comparison of all the seasonal SR deployment schedules at 100% coverage revealed that continuous use in all months of the year was the most effective for reducing new *Plasmodium* infections per capita after 2 years of intervention (median = 0.89 inf./pp./yr., SD = 0.15; Figure 6). The most effective single month deployment was in December (median = 1.39 inf./pp./yr., SD = 0.13), although single month deployments were the least effective overall. Six-month deployments in half-year and 3-2-3-month blocks varied substantially in effectiveness depending on which month the SR deployment began. The non-continuous deployments with the fewest annual infections per capita for each type of deployment were 8 months starting in September (Season-09) (median = 0.90 inf./pp./yr., SD = 0.14), 6 months starting in October (Half-10) (median = 1.03 inf./pp./yr., SD = 0.15), and 3-2-3-month blocks starting in September (Block-09) (median = 1.02 inf./pp./yr., SD = 0.18). The eight-month seasonal deployments starting in July through November all averted more infections than any of the six-month deployment schedules, while at least one 6-month deployment schedule averted more infections than eight-month seasonal deployments starting in December through June. Half-10 and Block-09 averted 89% as many infections per capita as continuous deployment, despite using only half as many products.

**Figure 7.**
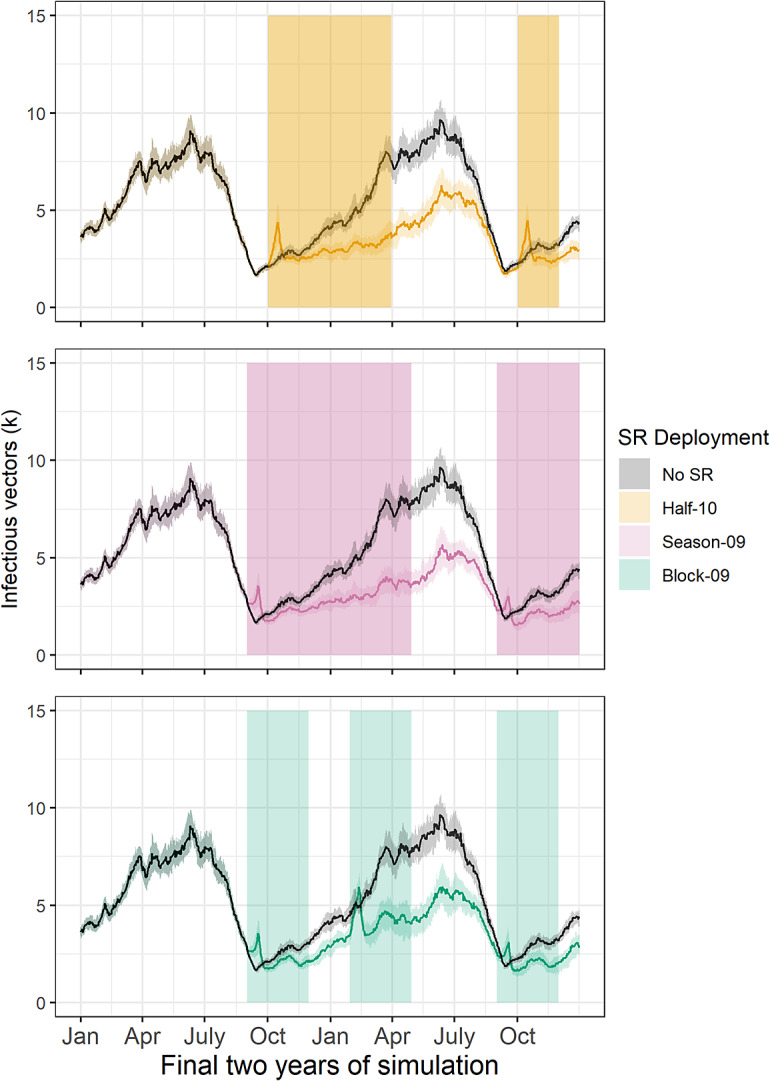
SR deployment schedules for Half-10, Season-09, and Block-09. Median infectious vectors from each deployment schedule are plotted against the median for No SR. Figure 7. long description.

The non-continuous deployment schedules with the greatest impact for their deployment type share 2 months in common (Figure 7). All three include SR deployment in October and November, when the EIR and number of infectious vectors start to rebound from minimums in September. This suggests that the most impactful time to use this vector control tool is when transmission is still low but increasing (October–December in this setting).

Both the Block-09 and Block-04 models included a block of SR deployment from September through November. The other block in each model covered February through April and April through May, respectively. In the second year of SR use, the Block-09 model averted 1.6 times as many new infections per capita as Block-04 did, with means of 0.62 and 0.40 infections averted per capita, respectively. This indicates that SRs had a greater impact on annual infection numbers when used during periods of lower, but increasing, transmission levels (February–April) than at near-peak transmission levels (April–June). This benefit may occur because SRs help to ‘flatten the curve’ early in the malaria cycle.

**Figure 8.**
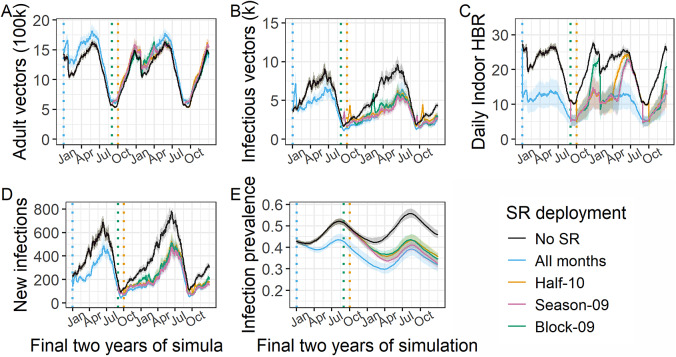
Median number of (a) adult vectors, (b) infectious vectors, (c) indoor per capita human biting rate, (d) new infections, and (e) infection prevalence across final 2 years of the simulation. Colours indicate SR deployment schedules. Dotted lines indicate the start of the first SR deployment for each schedule. Figure 8. long description.

The most effective non-continuous SR deployment schedules had similar seasonal patterns to the model with continuous SR deployment in all months of the year. Each of these deployment options exhibited an increase in the number of adult vectors and a decrease in the number of infectious vectors, the number of vectors feeding indoors, the number of new infections, and infection prevalence compared to the negative control (Figure 8). Notably, each model showed a short-lived spike in the number of infectious vectors after each introduction or reintroduction of SRs (Figure 8b). This happened only once in the All Months model and three times in the Block-09 model during the two-year intervention period. However, despite the increase in the vector population and the temporary spike in infectious vectors following SR deployment, the indoor human biting rate was consistently lower during SR deployment (Figure 8c).

**Figure 9.**
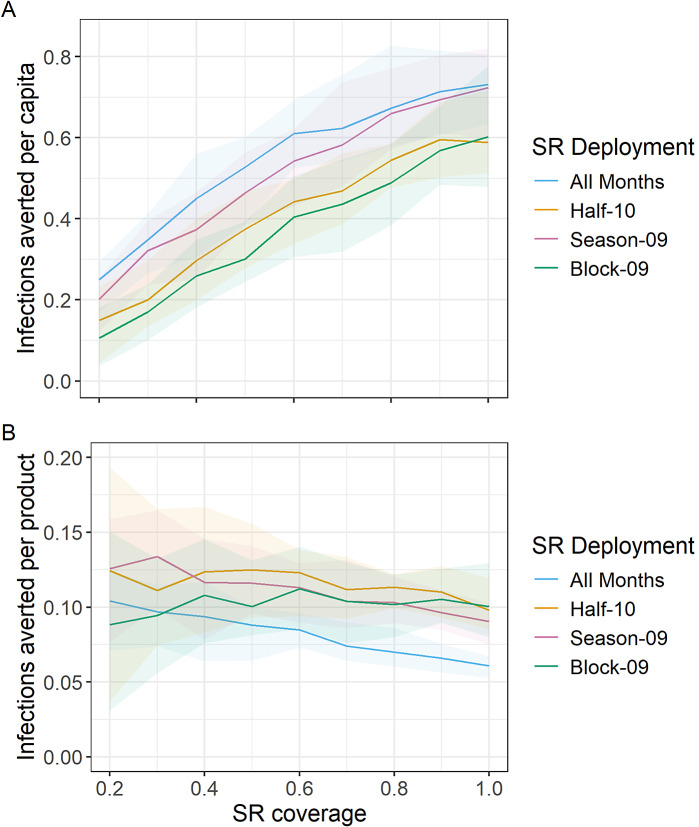
(a) Median infections averted per capita, and (b) infections averted per capita per product across SR coverages from 20% to 100%. Colours indicate SR deployment schedules. Figure 9. long description.

The most effective SR deployment schedules were assessed for their impact across a range of coverage levels from 10% to 100%. The year-round SR deployment averted the greatest number of infections across all levels of coverage, although the IQR overlapped with the median Season-09 impact and the IQRs of the Half-10 and Block-09 impacts (Figure 9a). The Season-09 deployment, which covers 8 months, consistently averted more infections than the six-month Half-10 and Block-09 impacts. All Months SR deployment averted a median 0.61 infections per capita at 60% coverage and 0.73 infections per capita at 100% coverage, achieving 84% of the maximum impact with 60% coverage. Half-10 SR deployment averted a median 0.44 infections per capita at 60% coverage and 0.59 infections per capita at 100% coverage, achieving 75% of the maximum impact with 60% coverage. The Season-09 deployment schedule also achieved 75% of the maximum impact with 60% coverage, while the Block-09 schedule achieved 67% of the maximum impact at 60% coverage. All Months SR use at 50% coverage averted slightly fewer infections (median = 0.53 pp) than 100% coverage with Half-10 or Block-09 deployments (medians = 0.59 and 0.60 pp) while using the same amount of product. Each of the optimal SR deployment schedules had a steady per product impact with increasing coverage (Figure 9b). Infections averted per capita per product decreased modestly with increasing coverage, except for the Block-09 deployment, which had the lowest impact per product at low coverage levels, but did not decline in efficiency as coverage levels increased. All Months deployment had the lowest per product impact except at the lowest coverage levels.

### Counterfactual scenario

**Figure 10.**
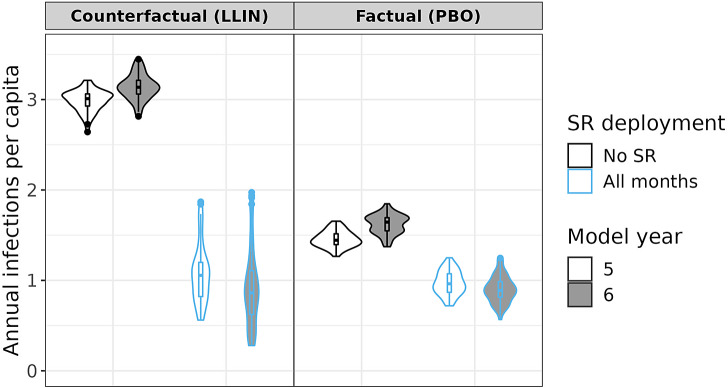
Infections averted per capita in model years five and six (intervention years one and two) under the counterfactual (pyrethroid-only LLIN) and factual (PBO bed net) scenarios. Black plots represent No SR. Blue plots represent All Months SR use at 100% coverage. Black dotted lines show the number of infections per capita from the corresponding baseline and placebo field data. Figure 10. long description.

Continuous SR deployment was also evaluated in a counterfactual scenario in which PBO bed nets were not distributed, but standard pyrethroid-only LLINs were used at coverage levels reported in the 2015 MIS. The bed net killing effect for pyrethroid-only LLINs was calibrated such that the model with no SR use would match the infection count from the baseline data collection of the SR field study (3.2 infections per capita per year) prior to the deployment of new PBO nets. The counterfactual model run with no SRs matched the baseline infection data fairly closely in years five (median = 2.99 infections pp./yr., SD = 0.12) and six (median = 3.12 infections pp./yr., SD = 0.14) of the simulations (Figure 10). The counterfactual model run with 100% SR coverage in all months had substantially fewer infections per capita in years five (1.07 infections pp./yr., SD = 0.32) and six (0.93 infections pp./yr., SD = 0.45). Under the counterfactual scenario, 2 years of continuous SR use averted about 4 infections per capita. The factual (PBO) model run with no SRs matched the placebo infection data fairly closely in years five (*M* = 1.46 infections pp./yr., SD = 0.09) and six (*M* = 1.62 infections pp./yr., SD = 0.11) of the simulations. The factual model run with 100% SR coverage in all months had fewer infections per capita in years five (0.97 infections pp./yr., SD = 0.13) and six (0.90 infections pp./yr., SD = 0.15). Two years of continuous SR use at 100% coverage under the factual scenario (PBO bed nets) averted about 1.2 infections per capita. These results suggest that SRs may have a greater impact when used under conditions like the counterfactual scenario with lower bed net coverage and higher transmission. Mean infections per capita with SR use in all months were similar under the counterfactual and factual scenarios despite large differences in infections with no SR use. Simulations of SR use under the counterfactual scenario produced infection estimates with twofold more variability than under the factual scenario.

## Discussion

Recent field studies indicate that SRs are an effective addition to the toolkit for reducing malaria transmission. This study aimed to evaluate seasonal SR deployment strategies for effectiveness and efficiency in averting new infections. As expected, continuous monthly SR deployment averted the most infections at a given coverage level. However, some non-continuous deployment options were more efficient, averting more infections per product. Non-continuous seasonal SR deployments averted more infections when started during periods of low and increasing transmission (September–December), as opposed to starting during the high transmission and rainy seasons. These results suggest that SRs will complement existing malaria control strategies.

The model used in this study was calibrated to match field data where continuous SR use at 100% coverage had 33% protective efficacy [22]. Model outputs from the first year of All Months SR deployment matched that value, reducing *Plasmodium* infections by 34% (IQR: −26% to 40%). Model outputs from the second year showed a 45% (IQR: −39% to 47%) reduction in new infections versus No SR, suggesting that the impact of SRs grows with additional years of use. Infection prevalence continued to decline in the second year of the intervention, suggesting that the continued use of SRs is driving longer-term reductions in the *Plasmodium* reservoir within the community and therefore exhibiting increased effectiveness over time. This is an intriguing result of our model simulations, as the field trial only followed cohorts of SR users for 1 year. Mean product application rates among study households in the spatial repellent group ranged from 82% to 100% over the course of the trial [22]. Our results indicate that the best deployment strategies are the same at 80% or 100% coverage whether measured as the highest infections averted per capita or per product. Because reaching high coverage levels may be difficult outside of a controlled trial setting, we also simulated SR deployments at lower coverage levels. The relative impacts of different deployment strategies are fairly consistent across coverage levels, with the top four strategies maintaining the same per-capita and per-product rankings at coverage levels from 20% to 80%. However, although the Block-09 deployment schedule was the most effective per-product at 100% coverage (by a narrow margin), it was less effective per-product than the Half-10 schedule at coverage levels below 100% and less effective than the Season-09 schedule at coverage levels below 60%. Therefore, the anticipated coverage level may have some influence on the best deployment strategy.

All Months deployment at 100% coverage performed the best in this model, but it may not be logistically feasible or cost effective to implement. Other simulated strategies were nearly as effective; Half-10 and Block-09 achieved 80%–82% the reduction in infections that All Months did while using half the number of SRs, and the eight-month Season-09 deployment achieved almost 99% of the reduction while only using 2/3 the amount of product. These two 6-month deployments prevented more infections per product than All Months at coverages above 30%. Notably, All Months at 50% coverage averted roughly the same number of infections per product as Half-10 and Block-09 at 100%. Each of these strategies would use the same number of SR products. This offers malaria control programmes the option of pursuing maximum coverage for half the year or moderate coverage for the whole year with similar anticipated effects.

Six-month deployment strategies beginning in different months had vastly different effects. The most effective half-year and 3-2-3 block deployments began coverage in September to December. The least effective half-year and 3-2-3 block deployments began coverage in March to June when the number of infectious vectors was already at or near its seasonal peak. Additionally, the most effective single month deployments were in November and December and the least effective were in March and April. These findings contradict the hypothesis that maximum protection against new infections would come from SR deployment during the major rainy season from April to June. This aligns with findings in a study that modelled IRS (effective 6 months) in Zimbabwe, where the intervention was most effective when carried out in August through December, offering protection months before peak transmission in March and April [29]. A possible explanation is that when transmission is highest, the EIR is so high that increasing protection in the home makes little difference in whether a person will get infected. Instead, interventions can have a greater impact on total infections by ‘flattening the curve’ when transmission is still low.

The best continuous half-year SR deployment beginning in October averted more infections than the best 3-2-3 block deployment beginning in September when SR coverages were 10%–90%. Unless SR coverage reaches >90%, it appears that the rebound in the number of infectious vectors that occurred during the two-month product break more than offset the additional infections prevented in the final 2 months of the 3-2-3 block deployment. The eight-month seasonal deployment starting in September averted more infections than either six-month deployment schedule and even averted more infections per product at low coverage levels (<40%), indicating that continuous monthly deployment during this period would be the most effective strategy. This eight-month deployment schedule averted almost as many infections as year-round SR coverage at coverage levels above 70% and averted more infections per product at all coverage levels. Therefore, an eight-month deployment strategy at relatively high coverage levels may provide the best balance of protection for a given population while also ensuring product availability for a larger population size.

In the counterfactual scenario with pyrethroid-only LLINs and lower bed net coverage (62%–77% depending on age), SRs averted nearly fourfold more infections than in the factual scenario with PBO bed nets at 95% coverage. The mean number of infections in the two scenarios was roughly the same, suggesting that in a community using pyrethroid-only LLINs, distributing SRs only might have a similar impact to distributing SRs and upgrading to PBO bed nets. However, further investigation of the counterfactual model assumptions is necessary before the model results should be used to inform intervention scenarios.

Our analysis focused on the deployment of an SR product that provides protection for 1 month before protective efficacy begins to wane. This matches the product profile of the Mosquito Shield™ SR product used in field trials in Kenya. SC Johnson has also now developed a longer-lasting SR product, Guardian™, which has demonstrated efficacy for up to 1 year [42]. Because the use of Guardian™ requires only 1/12 the amount of product for the same length of protection, it may prove more cost effective or easier to deploy at high coverage levels than an SR product that must be replaced monthly. However, our analysis shows that seasonal deployment schedules of a monthly SR product can be nearly as effective as a year-round deployment, so any assessment of which product to deploy will need to account for the timing and length of deployment needed in a given setting. The cost-effectiveness of a particular deployment strategy of either SR product is an important consideration that was beyond the scope of this manuscript as that will depend on the direct cost of each product as well as the magnitude of non-product administration costs that will vary by location and how deployment is administered. The Advancing Evidence for the Global Implementation of Spatial repellents (AEGIS) team is currently modelling the most cost-effective deployment strategies across Africa based on the local malaria burden and current LLIN usage rates by region.

Our model results suggest that SR products can be a beneficial addition to the roster of malaria control tools and that planning optimal deployment based on local epidemiology may help to devise cost-efficient implementation plans. This study highlights the utility of localized modelling for developing data-driven, site-specific interventions against infectious diseases.

## Supporting information

10.1017/S095026882610171X.sm001 Huwe et al. supplementary material Huwe et al. supplementary material

## Data Availability

The entomological and malaria prevalence data used to calibrate our model are openly available at https://github.com/mooresea/SR_deployment_malaria.

## Supplementary material

The supplementary material for this article can be found at http://doi.org/10.1017/S095026882610171X.

## Long descriptions

### Figure 2. Long description

The chart uses a vertical axis listing months from Jan to Dec. The months of Apr, May, Jun, Oct, and Nov are highlighted in blue to represent rainy seasons.

* The first column, labeled All, shows a solid green vertical bar indicating S R use for all twelve months.

* The top-middle panel, Single Month, shows a diagonal line of single green blocks moving from Jan at the top-left to Dec at the bottom-right.

* The top-right panel, Six Months, shows a thick diagonal band of six consecutive green blocks for each starting month, creating a staggered staircase pattern.

* The bottom-middle panel, Seasonal Eight Months, shows a wider diagonal band of eight consecutive green blocks for each starting month.

* The bottom-right panel, 3-2-3 Blocks, shows a fragmented pattern where each row contains three distinct green segments separated by white gaps, following a staggered diagonal flow from top-left to bottom-right.

Navigate back to Figure 2..

### Figure 3. Long description

The figure consists of five vertically stacked panels labeled A through E. The horizontal X axis for all panels represents time across the final two years of simulation, with tick marks for Jan, April, July, and Oct for each year.

Panel A. Rainfall in m m. The Y axis ranges from 0 to 15. The data shows a highly volatile, jagged line with seasonal peaks reaching approximately 10 to 15 m m around April and July, and troughs near 0 to 2 m m in October and January.

Panel B. Air temp in C. The Y axis ranges from 20 to 24. The data follows a sinusoidal wave pattern, peaking at 24 degrees Celsius around April and July and dipping to 21 degrees Celsius around October and January.

Panel C. Adult vectors k. The Y axis ranges from 0 to 1500. The line shows a smooth seasonal oscillation, peaking at approximately 1500 in July and dropping to a minimum of 500 in October.

Panel D. Infectious vectors k. The Y axis ranges from 0 to 15. The trend mirrors Panel C but at a lower scale, with a gradual increase from January to a peak of nearly 10 in July, followed by a sharp decline to approximately 2 in October.

Panel E. New infections. The Y axis ranges from 0 to 1000. The data shows a steady climb from 250 in January to a peak of approximately 750 in July, followed by a rapid decrease to a baseline of 100 in October.

In all panels, a solid black line represents the median, and a light gray shaded cloud represents the I Q R.

Navigate back to Figure 3..

### Figure 4. Long description

The Y-axis represents Proportion change from No S R, ranging from negative 1.00 to 0.25 with a dashed horizontal baseline at 0.00. The X-axis lists six categories. Each category has two violin plots: a white-filled plot for year 1 and a grey-filled plot for year 2.

* Adult vectors: Year 1 shows a slight increase above 0.00, while Year 2 shows a wider distribution centered slightly above 0.00 with a tail extending to negative 0.25.

* Infectious vectors: Both years show a significant decrease. Year 1 is centered around negative 0.25, and Year 2 is centered lower, around negative 0.45.

* E I R: Year 1 is centered around negative 0.25. Year 2 shows a broader distribution centered near negative 0.40.

* New infections: Year 1 is centered around negative 0.35. Year 2 is centered lower, near negative 0.45.

* New cases: Year 1 is centered around negative 0.25. Year 2 is centered lower, near negative 0.40.

* Infection Prevalence: Year 1 shows a small decrease centered near negative 0.15. Year 2 shows a larger decrease centered near negative 0.30.

Overall, Year 2 interventions consistently result in a greater proportional reduction across all metrics compared to Year 1, except for Adult vectors.

Navigate back to Figure 4..

### Figure 5. Long description

The figure consists of three vertically stacked panels sharing a common x-axis labeled Intervention year, ranging from 0 to 2.

Panel A at the top shows Rainfall in millimeters on the y-axis from 0 to 15. The data is a highly volatile black line with blue highlights, showing seasonal peaks roughly every 0.5 years, with the highest peaks reaching approximately 15 millimeters.

Panel B in the middle shows Infection prevalence on the y-axis from 0.0 to 0.6. A vertical dashed blue line at year 0 marks S R start. The No S R scenario, represented by a black line with a gray shaded confidence interval, shows a fluctuating but generally increasing trend from 0.4 to nearly 0.6. The All months scenario, represented by a blue line with a light blue shaded interval, shows a lower trend that dips after the S R start and fluctuates between 0.3 and 0.4.

Panel C at the bottom shows Prop. infections R D T plus on the y-axis from 0.0 to 0.5. Both the black No S R line and the blue All months line show clear seasonal oscillations. The black line peaks at approximately 0.35, while the blue line peaks slightly lower at approximately 0.32. Both lines follow a similar rhythmic pattern, with the blue line consistently lower than the black line after year 0.

A legend to the right of Panel B identifies the black line as No S R and the blue line as All months.

Navigate back to Figure 5..

### Figure 6. Long description

The y-axis represents the Proportion change in new infections, ranging from negative 1.00 to 0.00 in increments of 0.25. A black dashed line sits at 0.00. The x-axis is labeled Seasonal S R deployment and contains 49 categories. A blue dashed horizontal line at approximately negative 0.45 marks the average for the first category, All Months.

Moving left to right:

* The first category is All Months, showing a median change near negative 0.45.

* The next 12 categories are labeled 01 only through 12 only. These show the smallest reductions, with medians starting near 0.00 for 01 only and gradually dipping to around negative 0.15 by 12 only.

* The next 12 categories are Half-01 through Half-12. These show a downward trend in infection change, with Half-01 starting near negative 0.20 and Half-07 through Half-12 reaching deeper reductions between negative 0.30 and negative 0.40.

* The next 12 categories are Season-01 through Season-12. These show a U-shaped trend. Reductions deepen from Season-01 to Season-08, where the median reaches its lowest point near negative 0.50, before rising back toward negative 0.30 by Season-12.

* The final 12 categories are Block-01 through Block-12. These fluctuate with medians generally staying between negative 0.20 and negative 0.40.

Throughout the graph, individual black dots represent outliers, and vertical whiskers extend from each box to show the data range.

Navigate back to Figure 6..

### Figure 7. Long description

A multi-panel line graph with three vertical panels sharing a common X-axis and Y-axis. The Y-axis is labeled Infectious vectors k and ranges from 0 to 15. The X-axis represents the Final two years of simulation with tick marks for Jan, Apr, July, and Oct. A legend to the right identifies four S R Deployment categories: No S R in gray, Half-10 in yellow, Season-09 in pink, and Block-09 in teal. In all panels, a black line with a gray shaded confidence interval represents the No S R baseline, showing a bimodal seasonal peak reaching approximately 9k and 10k vectors.

* The top panel displays the Half-10 deployment. Two yellow shaded vertical blocks indicate the intervention periods. During these periods, the yellow data line deviates significantly below the black baseline, peaking at roughly 6k instead of 10k.

* The middle panel displays the Season-09 deployment. Two pink shaded vertical blocks represent longer intervention windows. The pink data line shows a sustained reduction compared to the baseline, maintaining levels between 2k and 5k during the peaks.

* The bottom panel displays the Block-09 deployment. Three teal shaded vertical blocks indicate intermittent intervention periods. The teal data line shows sharp drops during the shaded blocks and moderate increases in between, generally staying below 6k.

Navigate back to Figure 7..

### Figure 8. Long description

The figure consists of five line graphs labeled A through E and a legend. All graphs share a common X axis representing the final two years of simulation with monthly ticks from January to October. Vertical dotted lines in blue, green, and orange indicate the start of different S R deployment schedules.

Panel A, Adult vectors 100 k. The Y axis ranges from 0 to 20. All schedules show a seasonal bimodal peak. The No S R and All months schedules track closely at the highest levels, while other schedules show slight reductions during the second year.

Panel B, Infectious vectors k. The Y axis ranges from 0 to 15. The No S R line shows two major peaks. All S R deployment schedules significantly reduce the number of infectious vectors compared to the No S R control, with the All months schedule showing the lowest sustained levels.

Panel C, Daily Indoor H B R. The Y axis ranges from 0 to 30. The No S R line fluctuates between 10 and 28. S R deployment schedules generally lower the H B R, with the All months schedule maintaining the lowest rate near 10.

Panel D, New infections. The Y axis ranges from 0 to 800. Seasonal peaks are evident. All intervention schedules show a marked decrease in new infections compared to the No S R baseline, particularly in the second year peaks.

Panel E, Infection prevalence. The Y axis ranges from 0.2 to 0.6. The No S R line peaks at 0.55. All S R schedules result in lower prevalence, with the All months schedule reaching the lowest point near 0.3.

Legend. S R deployment categories include No S R in black, All months in light blue, Half-10 in orange, Season-09 in purple, and Block-09 in green.

Navigate back to Figure 8..

### Figure 9. Long description

Two vertically stacked line graphs, labeled A and B, share a common x-axis of S R coverage ranging from 0.2 to 1.0. A legend to the right of both graphs identifies four S R Deployment schedules: All Months in light blue, Half-10 in yellow-orange, Season-09 in pink-purple, and Block-09 in dark green.

* Panel A: The y-axis is Infections averted per capita, ranging from 0.0 to 0.8. All four deployment schedules show a positive, non-linear increase as S R coverage increases. All Months consistently shows the highest averted infections, starting at approximately 0.25 and rising to 0.75. Block-09 shows the lowest, starting near 0.1 and rising to 0.6. Shaded ribbons around each line indicate uncertainty intervals.

* Panel B: The y-axis is Infections averted per product, ranging from 0.00 to 0.20. In contrast to Panel A, these trends are relatively flat or slightly declining. Half-10 and Season-09 start with the highest efficiency near 0.13. All Months shows a steady decline from 0.10 at 0.2 coverage down to approximately 0.06 at 1.0 coverage. The lines converge and overlap significantly between 0.08 and 0.12 across the coverage range.

Navigate back to Figure 9..

### Figure 10. Long description

The two-panel violin plot has a shared Y-axis labeled Annual infections per capita ranging from 0 to 3.

Left Panel: Counterfactual L L I N.

* No S R deployment (black outlines): Model year 5 (white fill) shows a peak around 3.0. Model year 6 (grey fill) shows a higher peak around 3.2.

* All months S R deployment (blue outlines): Model year 5 (white fill) shows a significant drop to approximately 1.0. Model year 6 (grey fill) shows a similar drop with a wider distribution centered around 0.9.

Right Panel: Factual P B O.

* No S R deployment (black outlines): Model year 5 (white fill) is centered around 1.5. Model year 6 (grey fill) is slightly higher, centered around 1.7.

* All months S R deployment (blue outlines): Model year 5 (white fill) drops to approximately 1.0. Model year 6 (grey fill) shows a similar distribution centered around 0.9.

Legend:

* S R deployment: No S R (black square), All months (blue square).

* Model year: 5 (white square), 6 (grey square).

Navigate back to Figure 10..
