# Supplementary material for: Optimizing the impact of a novel spatial repellent on malaria incidence in Western Kenya
Source: Epidemiol Infect. 2026 Jun 8;154:e85. doi: 10.1017/S095026882610171X (PMC13312357; doi:10.1017/S095026882610171X)
Supplement: Huwe et al. supplementary material [file S095026882610171Xsup001.docx]

**SI Methods**

The three most important vector species in western Kenya are *Anopheles* *arabiensis, Anopheles* *gambiae,* and *Anopheles* *funestus.* To reproduce the relative seasonal abundance patterns of these three species, we fit a spline model to PMI Vectorlink data from 2016-2019 (1–4). The linear spline was then used to set the relative monthly larval habitat availability for each mosquito species (Fig. S1), with the absolute magnitude of larval habitat set via an additional parameter as described below. The indoor feeding fraction was set to 0.5 for *An. arabiensis,* and 0.98 for *An. gambiae* and *funestus*.


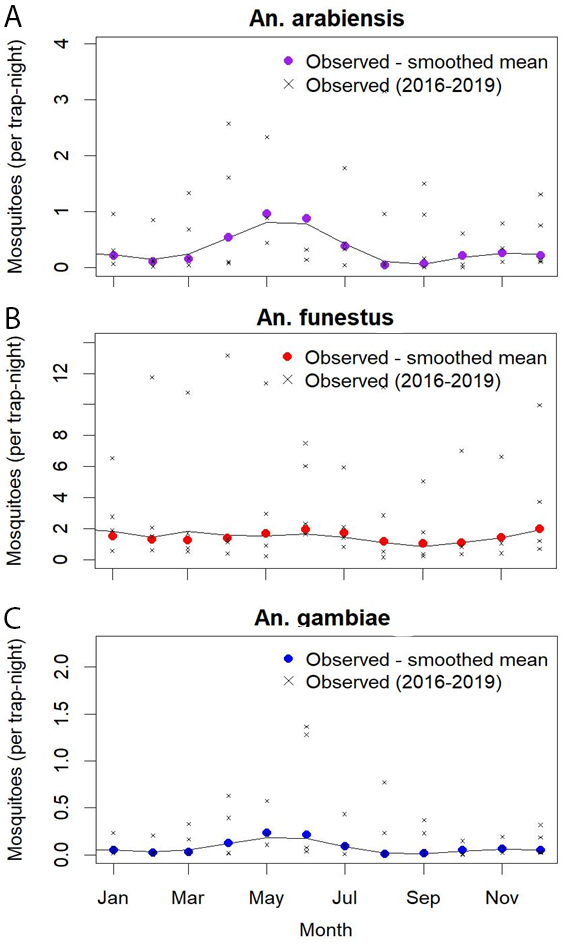


**Figure S1.** Monthly *Anopheles* collection data from 2016-2019 for (A) *An. arabiensis*, (B) *An. funestus*, and (C) *An. gambiae*. Xs represent monthly abundance data from a single year, while circles represent the mean abundance from 2016-2019. Lines represent the spline fit to the mean monthly data.

The model was fit to the mosquito abundance data from PMI, as well as age- and county-specific parasite prevalence from the 2015 Malaria Indicator Survey (MIS) (5). Model fitting was conducted using a maximum likelihood approach via a gradient ascent iterative optimization algorithm called OptimTool (6). We estimated the parameter that sets the maximum amount of larval habitat for each vector species and a scaling factor parameter for each of the six counties that adjusted vector abundance in that county relative to mean abundance. We initially ran 500 simulations with parameter values for each of the estimated parameters drawn using a sobol sequence. The likelihood of each simulation’s output was then estimated using (a) a beta binomial distribution to fit overall age-specific prevalence in the 0-4, 5-9, and 10-14 year old age groups, (b) a beta binomial distribution to fit county-specific prevalence in 0-14 year olds, and (c) Poisson distributions to fit monthly adult abundance of each the three main *Anopheles* species. The OptimTool algorithm was then used to draw starting parameter values for the next iteration of 500 simulations. This process was repeated for a total of 20 iterations, with the best fitting parameter set used for all subsequent analyses and simulations. The best-fit model simulations were able to reproduce both the infection prevalence in each of the six counties (Fig. S2), as well as the overall age-specific prevalences (Fig. S3).


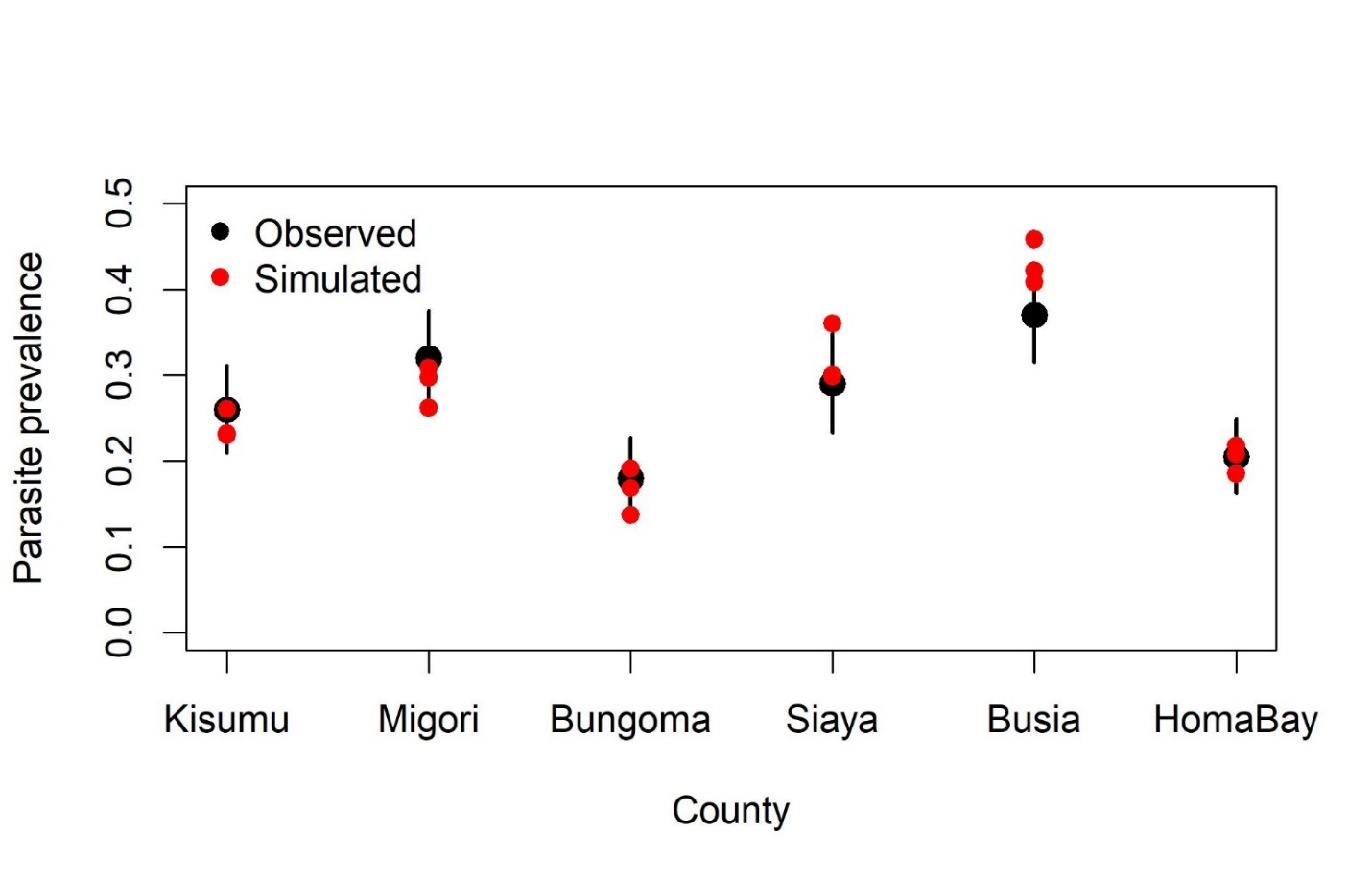


**Figure S2.** Best-fit model simulations of county-level parasite prevalence in 0-14 year olds. Observed values are from the 2015 Malaria Indicator Survey.


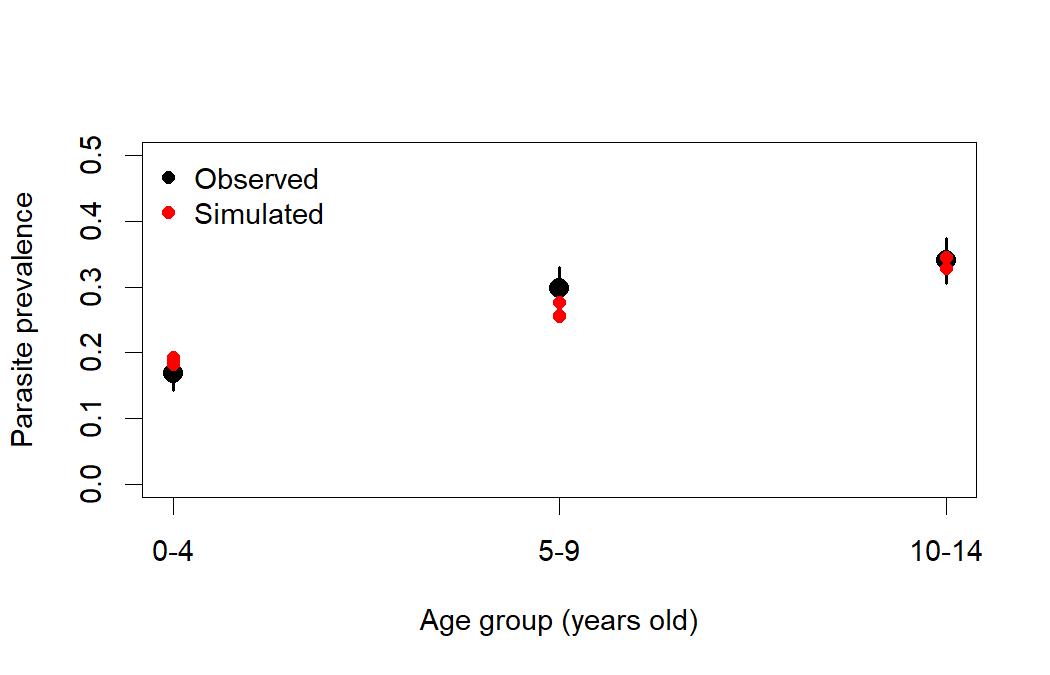


**Figure S3**. Best-fit model simulations of age-specific parasite prevalence in the 0-4, 5-9, and 10-14 year old age groups. Observed values are from the 2015 Malaria Indicator Survey.

Simulated vector abundance, infectious vectors, and new infections exhibited seasonal fluctuations, with infections spiking after the long rainy season as expected (Fig. S4). Seasonal cycles became stable after a four-year burn-in period. Further analysis was conducted on the outputs of simulation years five and six.

**Figure S4**. Model output data from six years of simulations with no spatial repellent (SR). Lines represent the median and clouds represent the IQR. Variables exhibit annual seasonality and become stable after three years of runtime.
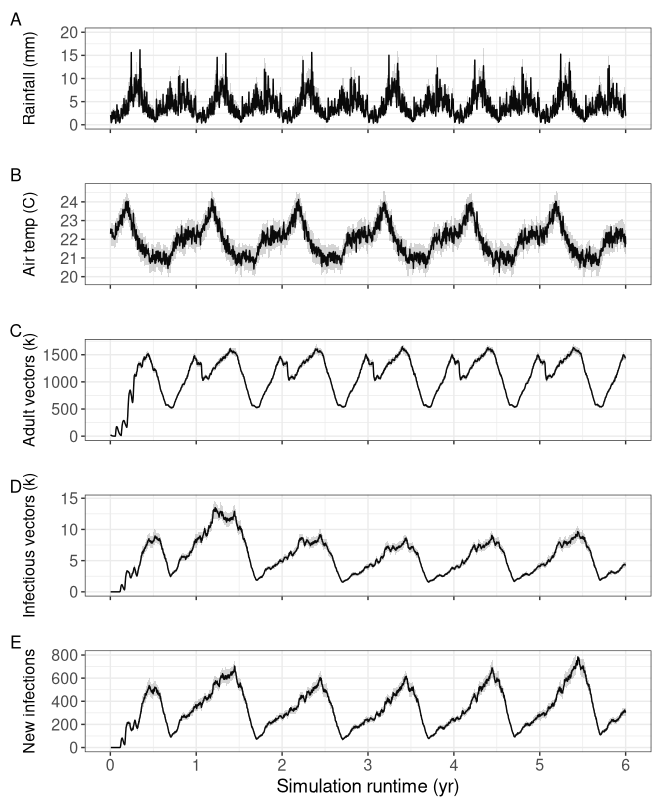


Bed nets were parameterized to fit data from the SR field trial published in 2024. After baseline data collection and before SR distribution for the field trial, a mass distribution of new piperonyl butoxide (PBO) bed nets was conducted in the study area. Therefore, PBO bed net effects were parameterized to fit data from the placebo arm of the field study during the implementation phase (1.42 infections/person/year) (7). PBO bed net coverage was set to 95% (7), and the initial blocking effect was set to 0.8. An appropriate initial killing effect was determined by running a sweep of bed net killing effect values from 0.05 to 1, and a parameter value of 0.45 provided the best match to field data (Fig. S5). Simulations run with these bed net parameters returned a mean incidence of 1.59 infections per capita in model year six.


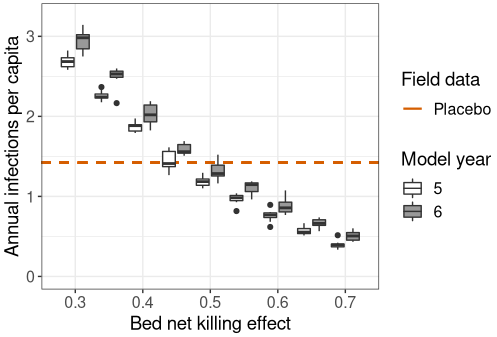


**Figure** **S5.** Annual infections per capita for model years 5 and 6 across possible bed net killing effects for PBO bed nets. The red dotted line indicates 1.4 annual infections per capita observed in the placebo data (7).

To assess a counterfactual scenario of SR deployment, pyrethroid-only LLIN effects were parameterized to fit data from the baseline of the field study (3.2 infections/person/year) (7). The LLIN blocking effect remained at 0.8, which represents an 80% probability that a host-seeking mosquito is prevented from reaching a host in a house with a bednet. LLIN coverage was set according to levels reported in the 2015 MIS (79%, 62% and 67% daily usage for ages 0-4, 5-14, and 15+) (8). Preliminary data indicate peak bednet usage rates during the baseline period were moderately higher than these estimates (90% for ages 0-4, 73% for ages 5+), but usage rates before 10PM and after 6AM were significantly lower (<25 – 75%), leading to overall usage rates comparable to the MIS-reported coverage levels.(9) An appropriate initial killing effect was determined by running a sweep of bed net killing effect values from 0.05 to 1, and a parameter value of 0.55 provided the best match to field data (Fig. S6). Simulations run with these bed net parameters resulted in a mean incidence of 3.16 infections per capita in model year six.


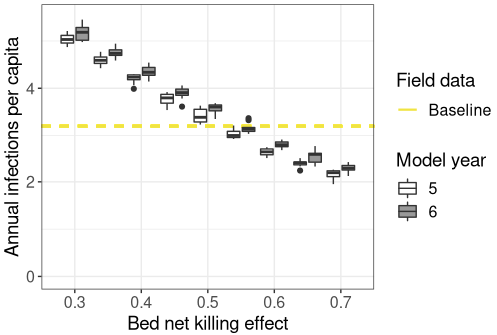


**Figure S6**. Annual infections per capita for model years 5 and 6 across possible bed net killing effects for pyrethroid-only LLINs. The yellow dotted line indicates 3.2 annual infections per capita observed in the baseline data (7).

The resulting killing effect estimates of 0.45 for PBO bed nets and 0.55 for pyrethroid-only LLINs is counterintuitive as PBO bed nets are anticipated to have similar or higher killing activity than pyrethroid-only LLINs. PBO bed nets contain a pyrethroid insecticide and piperonyl butoxide, a synergist that counteracts oxidase-dependent pyrethroid-resistance, restoring pyrethroid-susceptibility in many resistant mosquitoes (10). The unexpected result here may be due to the different coverage levels assumed for pyrethroid-only LLINs (62-79% depending on age group) and PBO bed nets (95%) during model fitting. PBO bed net coverage in the placebo arm of the trial may be overestimated, meaning that a higher killing effect would be required to fit the observed incidence in the placebo arm of the field trial. Alternatively, if the pyrethroid-only LLIN coverage at baseline was underestimated due to reliance on outdated data the killing effect required to match the baseline incidence rate would be smaller than estimated for LLINs. Another possible cause of this discrepancy is that we assumed the blocking effect of 80% was the same for LLINs and PBO nets, but it is possible that newer PBO bed nets have a stronger blocking effect than older LLINs. We assumed that the killing and blocking effects for both pyrethroid-only LLINs and PBO nets were constant and did not decay over time, in part because we did not know how long the pyrethroid-only LLINs had been in use. Not accounting for a decline in the effectiveness of the pyrthroid-only LLINs might also account for the discrepancy in the killing effect between the two bed net types.

*Spatial repellent effects*

Spatial repellent effects were parameterized to match the protective efficacy observed in the experimental arm of the field study (7). The observed 32.7% [CI 90%: 16.2 - 46.0%] protective efficacy against first-time malaria infection corresponded to an expected simulation output of 1.07 [CI 90%: 1.33 - 0.86] annual infections per capita. To determine appropriate SR parameters, simulations were run using 100 different combinations of SR blocking and killing effects ranging from 0 to 1 for each (Fig. S7A). A sobol sequence was used to generate combinations that covered the entire sample space evenly. SR coverage was set to 84% and new SRs were distributed every 30 days to model continuous deployment for one year as was done in the field trial (7). The mean number of new infections per capita for ten stochastic realizations and the standard deviation were calculated for each of the 100 parameter combinations (Fig. S7B). The likelihood of the point estimate 1.07 infections per capita falling in the simulated output range was calculated for each parameter combination (Fig. S7C). Likelihoods were normalized to a range from 0 to 1. Fifty parameter combinations were sampled from the distribution with replacement and with probabilities weighted according to the normalized likelihoods (Fig. S7D). The selected parameter combinations that fit the field data the best tended to have a roughly one to one ratio of SR blocking and killing effects.

We also assumed that the killing and blocking effects remained constant for the first 30 days, and then decayed exponentially with a decay rate of 1/30 per day.


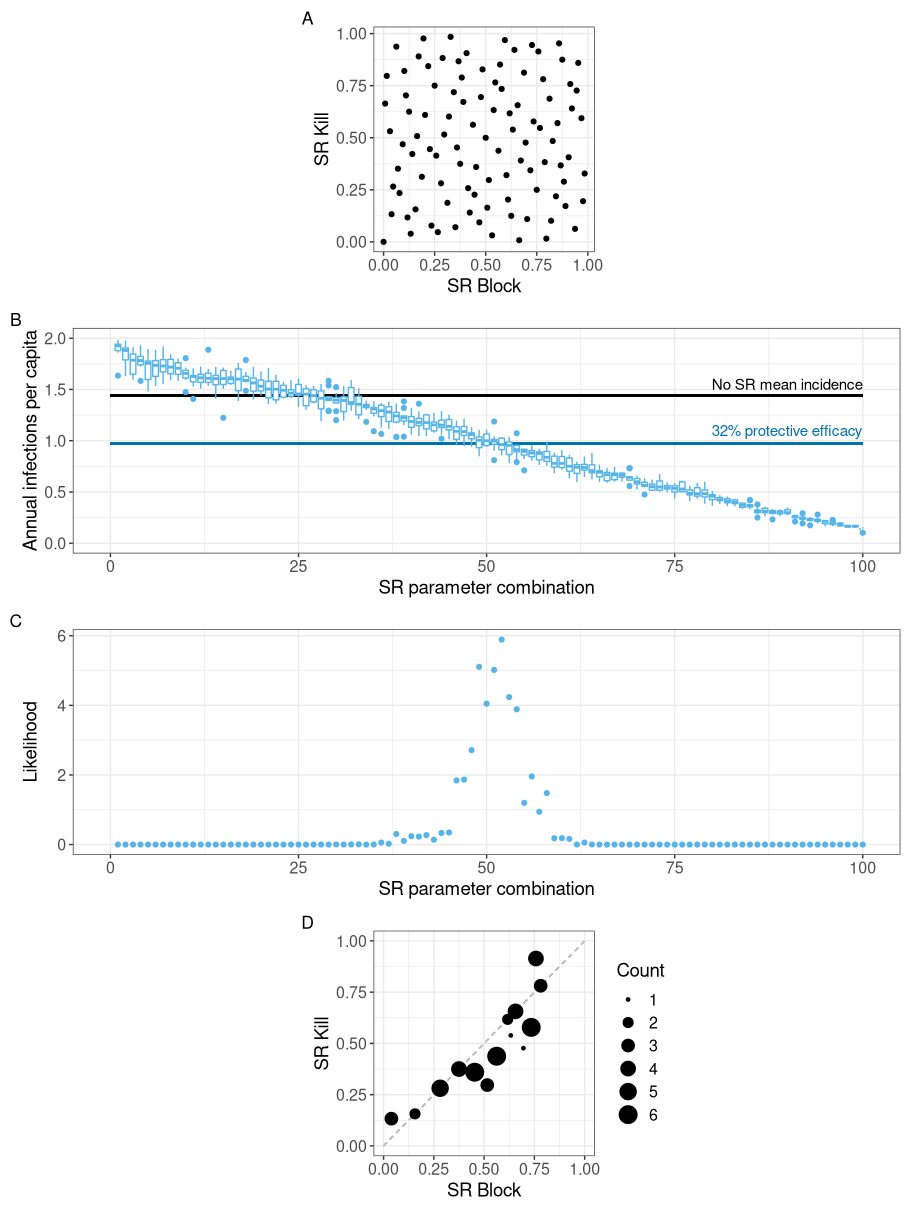


**Figure S7.** (A) SR parameter combinations to test. (B) Simulated annual infections per capita for each SR parameter combination tested. The black line indicates the mean incidence when the model was run with no SR. The blue line represents a 32% protective efficacy. (C) Likelihood that simulations using each SR parameter combination produce the number of infections expected in the case of 32% protective efficacy. (D) SR parameter combinations sampled from the likelihood distribution, weighted by frequency.

**References**

1. PMI Africa Indoor Residual Spraying Project (AIRS) Indoor Residual Spraying (IRS 2) Task Order Six. AIRS Kenya Annual Entomological Monitoring Report, December 2015 – September 2016. Bethesda, MD: Abt Associates, Inc.; 2017.

2. PMI Africa Indoor Residual Spraying Project (AIRS) Indoor Residual Spraying (IRS 2) Task Order Six. AIRS Kenya Entomological Monitoring Annual Report. December 2015 - September 2017. Rockville, MD: Abt Associates, Inc.; 2018.

3. PMI VectorLink Project. Annual Entomological Monitoring Report. October 2017- September 2018. Rockville, MD: The PMI VectorLink Project, Abt Associates Inc.; 2019.

4. PMI VectorLink Project. Kenya Annual Entomological Monitoring Report. October 2018- September 2019. Rockville, MD: The PMI VectorLink Project, Abt Associates Inc.; 2020.

5. National Malaria Control Programme. Kenya malaria indicator survey 2015. Nairobi, Kenya: NMCP, KNBS, and ICF International; 2016.

6. Moore SM, ten Bosch QA, Siraj AS, Soda KJ, España G, Campo A, et al. Local and regional dynamics of chikungunya virus transmission in Colombia: the role of mismatched spatial heterogeneity. BMC Med. 2018 Aug 30;16(1):152. doi:10.1186/s12916-018-1127-2

7. Ochomo EO, Gimnig JE, Awori Q, Abong’o B, Oria P, Ashitiba NK, et al. Effect of a spatial repellent on malaria incidence in an area of western Kenya characterised by high malaria transmission, insecticide resistance, and universal coverage of insecticide treated nets (part of the AEGIS Consortium): a cluster-randomised, controlled trial. The Lancet. 2025 Jan 11;405(10473):147–56. doi:10.1016/S0140-6736(24)02253-0 PubMed PMID: 39709979.

8. Bashir IM, Nyakoe N, Sande M van der. Targeting remaining pockets of malaria transmission in Kenya to hasten progress towards national elimination goals: an assessment of prevalence and risk factors in children from the Lake endemic region. Malar J. 2019 Dec;18(1):1. doi:10.1186/s12936-019-2876-x

9. Odero JI, Abong’o B, Moshi V, Ekodir S, Harvey SA, Ochomo E, et al. Early morning anopheline mosquito biting, a potential driver of malaria transmission in Busia County, western Kenya. Malar J. 2024 Mar 4;23(1):66. doi:10.1186/s12936-024-04893-3

10. Gleave^a^ K, Lissenden^a^ N, Chaplin M, Choi L, Ranson H. Piperonyl butoxide (PBO) combined with pyrethroids in insecticide‐treated nets to prevent malaria in Africa. Cochrane Database of Systematic Reviews. 2021;(5). doi:10.1002/14651858.CD012776.pub3
